# Fatigue Life Modelling of Steel Suspension Coil Springs Based on Wavelet Vibration Features Using Neuro-Fuzzy Methods

**DOI:** 10.3390/ma16062494

**Published:** 2023-03-21

**Authors:** C. H. Chin, S. Abdullah, S. S. K. Singh, A. K. Ariffin, D. Schramm

**Affiliations:** 1 Department of Mechanical and Manufacturing Engineering, Faculty of Engineering & Built Environment, Universiti Kebangsaan Malaysia, Bangi 43600, Selangor, Malaysia; chuinhao90@gmail.com (C.H.C.);; 2 Departmental Chair of Mechatronics, University of Duisburg-Essen, 47057 Duisburg, Germany

**Keywords:** multifractality, low-frequency energy, wavelet transform, ANFIS, durability

## Abstract

This study proposed wavelet-based approaches to characterise random vibration road excitations for durability prediction of coil springs. Conventional strain-life approaches require long computational time, while the accuracy of the vibration fatigue methods is unsatisfactory. It is therefore a necessity to establish an accurate fatigue life prediction model based on vibrational features. Wavelet-based methods were applied to determine the low-frequency energy and multifractality of road excitations. Strain-life models were applied for fatigue life evaluation from strain histories. ANFIS modelling was subsequently adopted to associate the vibration features with the fatigue life of coil springs. Results showed that the proposed wavelet-based methods were effective to determine the signal energy and multifractality of vibration signals. The established vibration-based models showed good fatigue life conservativity with a data survivability of more than 90%. The highest Pearson coefficient of 0.955 associated with the lowest RMSE of 0.660 was obtained by the Morrow-based model. It is suggested that the low-frequency energy and multifractality of the vibration signals can be used as fatigue-related features in life predictions of coil springs under random loading. Finally, the proposed model is an acceptable fatigue life prediction method based on vibration features, and it can reduce the dependency on strain data measurement.

## 1. Introduction

Durability analysis by associating the real operational loading to the fatigue damage calculation to determine the structural life is important in vehicle engineering. Some of the important elements of durability analysis include component geometry, material properties, and loading histories. Time domain approaches, such as stress-life and strain-life methods, are widely applied in current industry practices. However, the computational time of these approaches is long because a large loading sample is required to fulfil the statistical validity of the complex loading conditions. Therefore, other techniques based on fatigue loads such as frequency domain approaches [1,2,3], fatigue data editing [4], and fatigue load modelling [5] have been proposed to expedite the durability analysis. These techniques are often aided by signal processing tools such as the Fourier transform, power spectral analysis, and wavelet transform to extract essential fatigue-related features from the loading histories for modelling purposes. The fast Fourier transform (FFT) is most widely applied; however, it is not suitable for non-stationary signals due to its incapability to reveal inherent information. Although the short-time Fourier transform (STFT) could solve the shortcoming of FFT, it has the disadvantage of a constant time–frequency resolution. Later, wavelet transform was introduced as a solution to the resolution problem of STFT, which makes it an appropriate method for a wide variety of signals [6].

Wavelet-based signal processing methods are emerging as one of the popular analysis tools in fatigue analysis. Apart from time–frequency analysis, wavelet theory had been extended to singularity analysis that had been extensively used in fault detection or structural health monitoring [7,8,9]. Mohanraj et al. [8] and Zhou et al. [10] proposed the use of Hölder exponents to characterise the singularities in loading signals of milling tools to monitor the tools’ condition. Singularities in the loading signal are regarded as the locations where abrupt and larges changes in amplitude or frequency are detected. These changes are often regarded as transient events and associated with high fatigue damage. Previous works by Chin et al. [9,11] demonstrated that singularities extracted from the vibration loading signals of coil springs could effectively represent the low-frequency features of the signal which contributed to major fatigue damage. Furthermore, wavelet analysis also developed into multifractal theories including the wavelet leaders method [12]. Multifractality refers to the property of complex systems where different parts of the system exhibit varying degrees of scaling behaviour. In signal processing applications, multifractality quantifies the complexity of the signals based on the scaling properties. Multifractal analysis becomes relevant to the fatigue analysis of the suspension component as the road profiles naturally exhibit multifractal behaviour because they often have a complex structure that includes multiple scales of roughness [13,14]. Previous works by Chin et al. [9,14,15] had established one-to-one relationships between the singularity-based low-frequency energy and the multifractality of road excitations to the durability of coil springs using a wavelet-based analysis. In this study, both the wavelet-based singularity and multifractal parameters were integrated to characterize the road-excited loading signals for more accurate fatigue life prediction of suspension coil springs.

In recent years, machine-learning-based data analysis techniques have been proven to be powerful tools to analyse fatigue data related to complex real-life loading conditions [16]. This is because the fatigue life data often exhibit highly non-linear behaviours, which render fatigue data modelling using classical regression tools difficult. Therefore, machine-learning methods such as artificial neuro-networks (ANN) and neuro-fuzzy methods are applied in durability modelling. Kong et al. [17] proposed a model that bridged the ride comfort and durability of coil springs with various spring designs using the ANN method. However, the study did not consider the loading parameters in the durability model, even though the fatigue damage is often directly related to the loading. Moreover, the ANN method is also a black-box modelling technique, which did not provide sufficient reasoning power to explain the relationship between the inputs and output. Apart from ANN, the adaptive neuro-fuzzy inference system (ANFIS) was extensively used for the modelling of complex fatigue life data obtained from fatigue loads [18]. Unlike ANN, ANFIS technique has good reasoning power of fuzzy theory so that the user could comprehend the relationship of inputs and outputs. Machine-learning approaches have a better capability to fit highly non-linear data and improve the accuracy in predicting fatigue life compared to the classical regression approaches.

However, to the extent of the authors’ knowledge, a machine-learning fatigue life prediction model for suspension coil springs using vibrational features is still lacking. Thus, this study aims to establish a fatigue life prediction model based on vibrational features of random loading signals. Due to the high computational time of conventional strain-life approaches, researchers are looking for alternative approaches to model the durability performance of coil springs using the fatigue-related features of loading histories to achieve faster and more accurate durability prediction. Using signal processing techniques, important features related to fatigue life are determined from random loading signals of coil springs. Then, a machine-learning-based technique is used to establish the relationship between the vibrational features and the fatigue life. The established model is expected to provide a faster and more accurate alternative to predict fatigue life from vibrational loading of coil springs under various real-life road conditions. It is hypothesised that if the vibrational features have a close relationship with the fatigue life, then a fatigue life prediction model can be established.

## 2. Methodology

**Figure 1:**
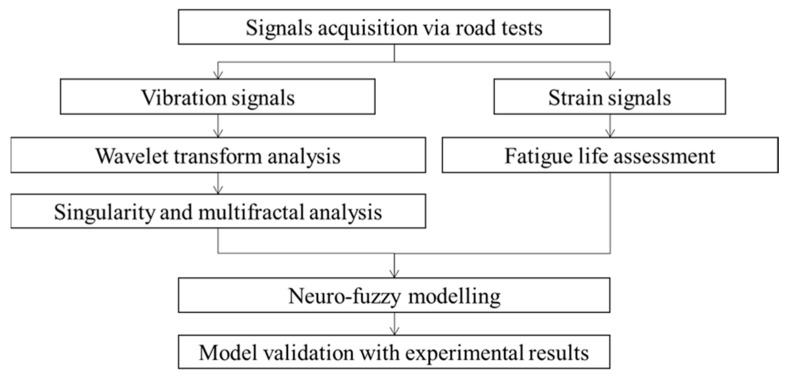
Methodology process flow to establish vibration-based durability prediction model.

Figure 1 depicts the process flow of the methodology to establish a vibration-based durability prediction model for coil springs under random road excitations. The study mainly focused on the fatigue feature extraction from the vibration signals via wavelet-based signal processing methods. Furthermore, the fatigue features were then related with the durability of the coil spring through neuro-fuzzy modelling.

### 2.1. Road Tests for Loading Data Acquisitions

**Figure 2:**
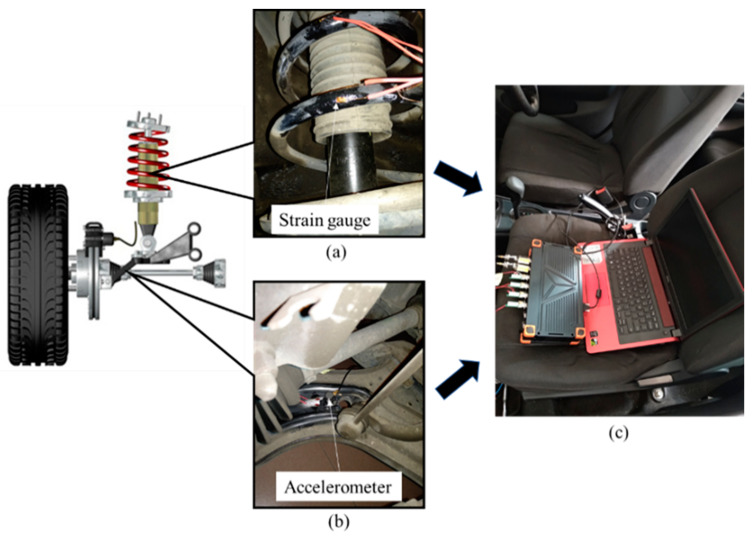
Instrumental setup for signal acquisition: (**a**) strain gauge, (**b**) accelerometer, and (**c**) data logger.

**Figure 3:**
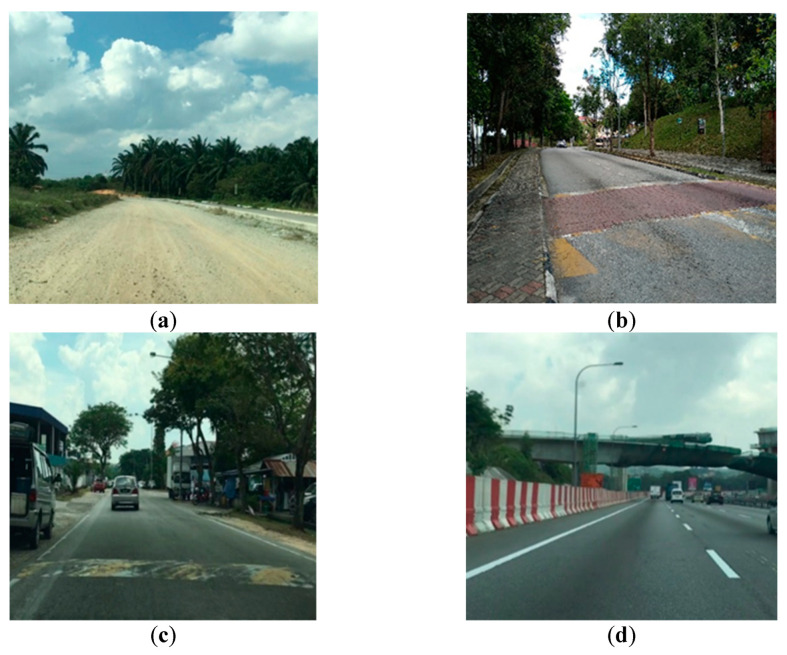
Road surface conditions chosen for loading data measurements: (**a**) rural road, (**b**) university campus, (**c**) industrial road, and (**d**) highway.

Road tests were carried out on different types of road terrains to measure the vibration and strain signals of the coil spring. Figure 2 shows the installation of the sensors and data acquisition instrument to measure and record the loading signals. A uniaxial strain gauge with 2 mm gauge length was firmly attached to the surface of the coil spring at the position near the hotspot, which was determined via finite element analysis. The procedures of the finite element analysis related to the boundary conditions can be found in [19]. This was to ensure that the maximum strain that contributed to high fatigue damage was measured. Furthermore, a uniaxial accelerometer was located on the lower control arm to measure the vertical acceleration resulting from the wheel motion as the wheel vertically bounced from road excitations. The acceleration signals could also represent the vibration loading of the suspension system as the vibrations of the suspension system were mainly contributed by the road surface roughness. The sampling frequency was set at 500 Hz, as suggested in previous studies [20,21], to avoid significant information loss due to under-sampling. Eighty seconds of the strain and vibration signals were simultaneously and repeatedly recorded with the data logger under various road conditions, namely in rural areas, on a university campus ground, in industrial areas, and on the highway. This is important so as to collect loading signals with various behaviours. The car velocity in the university campus, industrial, and rural areas was maintained at 20–40 km/h, while on the highway, it was 70–80 km/h [22]. The road tests were repeated to collect 30 loading signals from each type of road. Figure 3 shows the road conditions chosen for the road tests.

### 2.2. Vibration and Strain Signals Characterisations

Statistical parameters including mean, root-mean-square (RMS), skewness, and kurtosis of acquired signals were characterised. These statistical parameters revealed general behaviours of the signals and were highly correlated to the durability properties of the signals. The mean value provided information about the tensile or compressive nature of the loading. RMS represented the dispersion of data amplitude and energy content of the signals [23]. The skewness value was used to examine the symmetry of data distribution and to project extreme data in the signal. The kurtosis, on the other hand, was related to the extreme data and a measure of deviation of the data distribution from the Gaussian distribution [24]. Researchers [1,25] supported that loading with a lot of extreme data or large amplitudes can lead to a higher kurtosis value and is always associated with higher fatigue damage.

The continuous wavelet transform (CWT) was conducted to reveal the loading characteristics in the time–frequency domain. CWT is a time–frequency domain analysis, and it is effective in detecting transients in time series. It is also a process of breaking the time series into smaller windows with different translation and scale using the mother wavelet function $\psi_{s,u}\left(t\right)$, which is represented by

(1) $$\psi_{s,u}\left(t\right)=\frac{1}{\sqrt{s}}\psi (\frac{t-u}{s})$$

where *s* is the scaling parameter and *u* is the shift parameter. The CWT of a time series obtains wavelet coefficients and can be mathematically expressed as follows [26]:

(2) $$W_{\psi }f\left(s,u\right)=\langle f\left(t\right),\psi_{s,u}\left(t\right)\rangle =\frac{1}{\sqrt{s}}\int_{-\infty }^{\infty }f\left(t\right)\psi^{*}(\frac{t-u}{s})dt$$

where $\psi^{*}$ denotes the complex conjugate of the mother wavelet function.

### 2.3. Determination of Low-Frequency Energy

In a great deal of research, the energy content in loading histories is correlated to high fatigue damage that is associated with large amplitude cycles [27,28]. The road excitations, which cause fatigue damage in suspension components, are often low-frequency excitations of less than 20 Hz [29]. One of the main challenges in signal energy characterisation for durability analysis is the undesired noises in the signals, often associated with high frequency, caused by instrumental factors during signal acquisition. Hence, a denoising processing prior to the energy characterisation is required to eliminate the high-frequency noises.

The low-frequency energy of vibration signals was determined using the singularity method. In the durability context, singularities in loading histories are often associated with high-amplitude events corresponding to large fatigue damage [9]. In this study, singularities in the vibration signals were identified using Hölder continuity, such that

(3) $$\left|f\left(x\right)-P\left(x-x_{0}\right)\right|\leq C{\left|x-x_{0}\right|}^{\alpha }$$

where *f*(*x*) is a time function, constant *C* is larger than zero, and a polynomial *P* has degree *m* < *α*. The pointwise Hölder exponent (HE) is the supremum of *α* as defined above, which represents the local regularity of a time series.

The CWT, as expressed in Equation (2), was performed on the vibration signals. This was because CWT could effectively reveal the transients or irregular behaviours in the time signal as wavelet coefficients with a large magnitude [7]. These wavelet coefficients were later expressed as the wavelet transform modulus maxima (WTMM). The singularities were detected at the locations where the WTMMs converged at a finer scale.

To determine the singularities in the low-frequency range, the choice of the mother wavelet function is important. A mother wavelet function with a low vanishing moment is recommended to obtain low-frequency singularities. Recent studies [9,10] found that the second-order derivative of the Gaussian (DOG) wavelet is appropriate for singularity analysis because of its well-defined vanishing moments corresponding to its order of derivative. A DOG wavelet with *m* derivative order is given as

(4) $$\psi^{\left(m\right)}\left(t\right)=\frac{{\left(-1\right)}^{m+1}}{\sqrt{\Gamma \left(m+\frac{1}{2}\right)}}\frac{d^{m}}{dt^{m}}\left(e^{-\frac{t^{2}}{2}}\right)$$

where *m* represents the order of derivative. The second-order DOG wavelet has *m* = 2 and vanishing moments of two.

Subsequently, the obtained singularities were reconstructed into vibration signals at 500 Hz using the linear interpolation method [9]. The power spectral densities (PSD) of the reconstructed signals were computed using the following equation:

(5) $$P\left(\omega \right)=\frac{1}{N}{\left|\hat{f}\left(\omega \right)\right|}^{2}$$

where $P\left(\omega \right)$ denotes PSD of time series, $\hat{f}\left(\omega \right)$ is the Fourier transform of the time series, and *N* is the number of data points. Finally, the low-frequency energy (*E*) was computed from the PSDs of the reconstructed signals, such that

(6) $$E_{PSD}=\int_{0}^{\infty }P\left(\omega \right)\;d\omega$$

### 2.4. Wavelet Leaders Method to Determine Multifractality of Vibration Signals

Apart from the low-frequency energy, multifractality was another fatigue-related feature extracted from the vibration signals. Quan et al. [13] proved that the road pavement profile exhibits obvious multifractal properties that are related to the road surface roughness. It was also found in recent studies [14,15] that road multifractality has a non-linear relationship with the durability of coil springs. Hence, the multifractality of vibration signals was proposed as a fatigue life prediction parameter in this study.

Wavelet leaders (WL) multifractal formalism was proposed for multifractal analysis of vibration signals in this study. The WL method is a wavelet-based multifractal numerical method, in which the discrete wavelet transform (DWT) is used. The DWT-based theory of the WL method offers better computational efficiency for non-stationary signals compared to other multifractal formalism methods [12,30]. The detailed theoretical framework of WL multifractal formalism can be found in [14,15]. In this study, a Daubechies 2 (*db2*) wavelet was used for the WL multifractal analysis because the *db2* wavelet has two vanishing moments that are suitable for vibration signals [15,31]. Through WL multifractal formalism, a multifractal spectrum was obtained from each vibration signal. A multifractal spectrum consisted of two axes representing the fractal dimension *D*(*h*) and Hölder exponent (*h*). The scaling properties in a multifractal signal were represented by the Hölder exponent. Subsequently, multifractality was computed from the spectrum width (Δ*h*).

### 2.5. Fatigue Life Assessment with Strain-Life Approaches

By referring to the results of static load FEA of the coil spring in previous work [19], the highest von Mises stress (1136 MPa) was found to have exceeded two-thirds of the yield strength (1487 MPa). This suggested a high possibility of plastic deformation in the coil spring under extreme loading condition. Moreover, plastic strain can also be dominant in coil springs because of the small geometry, and thus the strain-life approaches are appropriate for the durability analysis [32]. The fatigue life was calculated from the strain histories of coil spring. Cycle counting using the rainflow algorithm was conducted before the fatigue life calculation. The rainflow algorithm is a widely applied cycle-counting method in current industrial practices due to its accurate results. Subsequently, the strain-life models, namely the Coffin–Manson, Morrow, and Smith–Watson–Topper (SWT) models, were employed for the fatigue life calculation, such that [33]

(7) $$\varepsilon_{a}=\frac{\sigma_{f}^{\prime }}{E}{\left(2N_{f}\right)}^{b}+\varepsilon_{f}^{\prime }.{\left(2N_{f}\right)}^{c}$$

(8) $$\varepsilon_{a}=\frac{\left(\sigma_{f}^{\prime }-\sigma_{m}\right){\left(2N_{f}\right)}^{b}}{E}+\varepsilon_{f}^{\prime }.{\left(2N_{f}\right)}^{c}$$

(9) $$\sigma_{max}\varepsilon_{a}=\frac{{\left(\sigma_{f}^{\prime }\right)}^{2}}{E}{\left(2N_{f}\right)}^{2b}+\sigma_{f}^{\prime }\varepsilon_{f}^{\prime }.{\left(2N_{f}\right)}^{b+c}$$

where $\varepsilon_{a}$ is the alternating strain amplitude, *E* is the modulus of elasticity, $\sigma_{f}^{\prime }$ is the fatigue strength coefficient, *b* is the fatigue strength exponent, $\varepsilon_{f}^{\prime }$ is the fatigue ductility coefficient, *c* is the fatigue ductility exponent, $\sigma_{max}=\sigma_{m}+\sigma_{a}$, $\sigma_{m}$ is the mean stress, and 2*N_f_* is the load reversals to failure at specific stress amplitude.

Fatigue damage *D_i_* caused by one fatigue cycle at a specific stress level can be expressed as

(10) $$D_{i}=\frac{1}{N_{fi}}$$

where *N_fi_* is the cycles to failure at a specific stress level.

Lastly, the Palmgren–Miner rule was utilised to calculate the total fatigue damage *D* in the loading block, such that

(11) $$D=\sum \frac{n_{i}}{N_{fi}}$$

where *n_i_* is the counted fatigue cycles at a specific strain range in the loading block. It is generally accepted that the combination of the rainflow counting and the Palmgren–Miner rule provided the most accurate results for variable-amplitude loading [34].

### 2.6. Adaptive Neuro-Fuzzy Inference Systems (ANFIS) Modelling

This study employed a machine-learning approach to establish a non-linear relationship between the vibration-based features and the fatigue life of coil springs. Previous studies [9,14] showed that the low-frequency energy and multifractality of vibration signals that represent the road excitations have non-linear relationships with the fatigue life of coil springs. Therefore, the ANFIS technique was used to establish the durability prediction model based on low-frequency energy and multifractality. The ANFIS is an integrated machine-learning technique of artificial neuro-network and fuzzy logic, which has exceptional capability in analysing non-linear fatigue data and offers additional reasoning power to the user to understand the behaviour of input parameters. The ANFIS modelling technique has been successfully applied to model highly non-linear fatigue data [35,36].

As the main objective of this study is to establish a durability prediction model for suspension coil springs based on vibration features, a two-inputs-one-output Takagi–Sugeno ANFIS network was then developed. The two inputs were the low-frequency energy and multifractality determined from the vibration signals, while the output was the fatigue life of the coil spring. The Takagi–Sugeno ANFIS network structure is a five-layer structure, and it has been extensively used because of its high robustness [37]. A Gaussian-type MF was used because it is continuous and is represented by two parameters only [38]. The learning process of ANFIS modelling was governed by a feedforward–backpropagation algorithm. This algorithm enabled the self-learning ability of the ANFIS structure to better adapt to the input data [39].

The dataset consisted of low-frequency energy, multifractality, and fatigue life was prepared for ANFIS modelling. Prior to the ANFIS modelling, the fatigue lives were converted into a base-10 logarithm scale for appropriate fitting [39]. A dataset consisting of 220 data was used for the modelling, as shown in Appendix A. Researchers [35,40] recommended a sample size above 100 samples for machine-learning-based durability modelling so that the samples are statistically representative of the real loading condition. The input dataset was divided into training and testing data at a ratio of 9:1. A testing dataset is necessary to avoid overfitting, a situation in which the model is too adapted to the input data. Overfitting can cause serious errors in the predicted results when fresh input data is introduced to the trained model. Before the model was trained, the subtractive clustering method (SCM) was used to divide the data points into several clusters. SCM is a fuzzy clustering method to determine the cluster centres in a given set of data [41]. The number of clusters represents the number of fuzzy rules involved in the ANFIS modelling. Fuzzy rules are a series of IF–THEN rules that relate the input and output MFs and can be expressed as follows:

(12) $$IF\;\;\;\;\;\;\;\;\;\;\;\;\;\;\;\;\begin{array}{cc} x_{1}=A_{j} & \;j=1,\;\ldots \;,S_{1} \end{array}$$

(13) $$\;AND\;\;\;\;\;\;\;\;\;\;\;\begin{array}{cc} x_{2}=B_{k} & k=1,\;\ldots \;,S_{2} \end{array}$$

(14) $$THEN\begin{array}{cc} \;\;\;\;\;\;\;\;\;\;\;\;f_{i}=o_{i}x_{1}+p_{i}x_{2}+q_{i}\; & i=1,\;\ldots \;,S_{1}\times S_{2} \end{array}$$

where *A* and *B* are the fuzzy membership set of the input variables *x*_1_ and *x*_2_, respectively. *S*_1_ and *S*_2_ are the numbers of MFs, and *f* is the linear consequence function with *o*, *p*, and *q* as the linear coefficients. In ANFIS modelling, it is important to determine the optimised fuzzy rule number. A large number of fuzzy rules offer better prediction accuracy but also significantly increase the computational load and the risk of overfitting. In this study, the ANFIS models with two to seven fuzzy rules were trained, and the model with the least error was selected.

### 2.7. Comparison of ANFIS-Based Durability Prediction Model with Strain-Life Approaches

Once the ANFIS model was trained, the fatigue lives predicted by the model were validated with the experimental fatigue lives. A life conservative analysis was performed by correlating the predicted and experimental fatigue lives within the boundary of 1:2 and 2:1. This analysis was important to ensure that the ANFIS-based prediction model provided acceptable fatigue life predictions with a safety factor of two. In addition, the predicted and experimental fatigue lives were correlated to determine the Pearson correlation coefficient (*r*). An *r* value close to one signifies a better correlation between the predicted and experimental fatigue lives and, thus, a more accurate ANFIS model. Lastly, a 95% confidence interval data survival analysis was added to test the statistical validity of that the predicted fatigue lives.

## 3. Results and Discussion

This section discusses the findings of analyses which included the characterisation of vibration signals, fatigue life assessment, and neuro-fuzzy modelling.

### 3.1. Vibration Signal Characterisation

**Figure 4:**
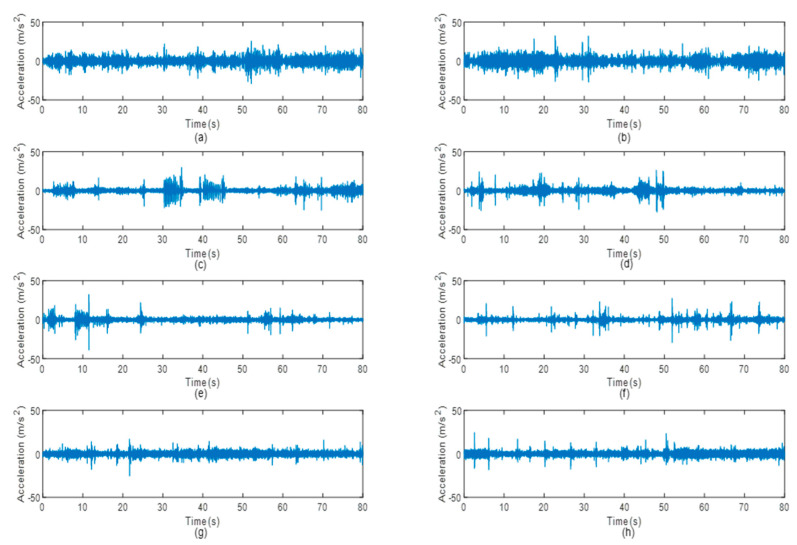
Vibration signals of the suspension system acquired under various road conditions: (**a**) Rural 1, (**b**) Rural 2, (**c**) Campus 1, (**d**) Campus 2, (**e**) Industrial 1, (**f**) Industrial 2, (**g**) Highway 1, and (**h**) Highway 2.

Vibration signals were acquired during the road tests under various road surfaces, as illustrated in Figure 4. The vibration signals acquired in the rural areas were found to have the most intense excitations, which were associated with several high-amplitude cycles. This was due to the rough surface of the unpaved rural roads. The highway, on the contrary, had a smooth surface profile and resulted in very minimal high-amplitude cycles in the vibration signals. The high-amplitude cycles indicated strong road excitations due to bumps, potholes, or rough road surfaces that resulted in significant fatigue deterioration.

Table 1 depicts the statistical properties of the vibration signals. It should be noted that the vibration signals measured by the accelerometer inherently had a zero mean, and therefore, the mean values of the signals are not shown. Due to the rough surfaces of the rural roads, ‘Rural 1’ and ‘Rural 2’ signals had the largest RMS values, indicating that the signals contained the largest energy. Meanwhile, the ‘Highway 1’ signal had the lowest RMS value of 2.14 m/s^2^ as the wheel experienced the least excitation when travelling on the smooth highway surface. When the signals have higher RMS values, the suspension system is expected to suffer more fatigue damage due to the higher energy. On the other hand, the skewness values of the vibration signals were found to be close to zero, indicating a perfect symmetry of the data distribution. Finally, the kurtosis values of larger than three indicated that the vibration signals behaved non-stationary. The kurtosis also represented the ‘peakedness’ of data distribution that was related to extreme data in a signal. The signals collected on campus grounds and in the industrial areas had very high kurtosis owing to the high-amplitude events with transient characteristics. These events eventually contributed to sharp data distribution and, hence, a higher kurtosis value. Previous studies [23,24] demonstrated that high-kurtosis loading signals are likely to inflict more fatigue damage to the structure.

**Figure 5:**
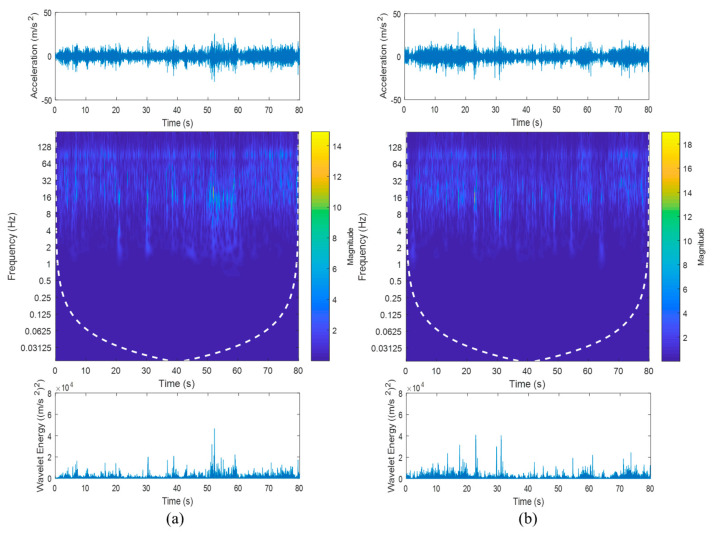

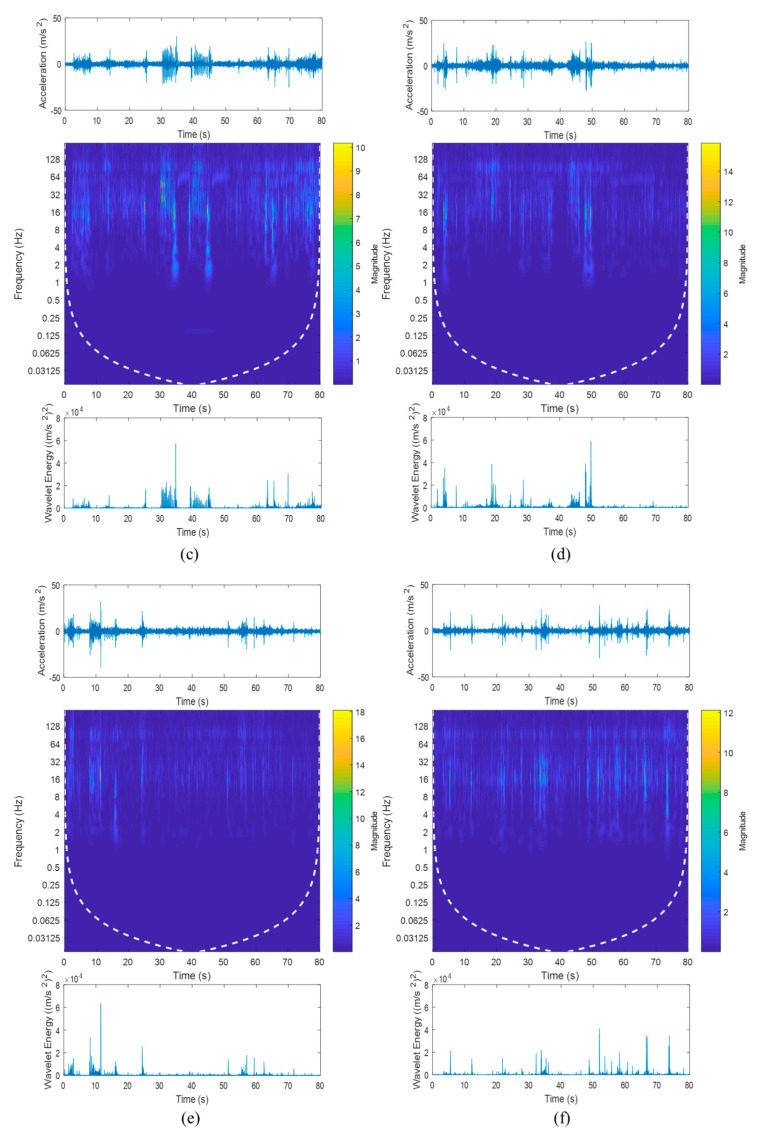

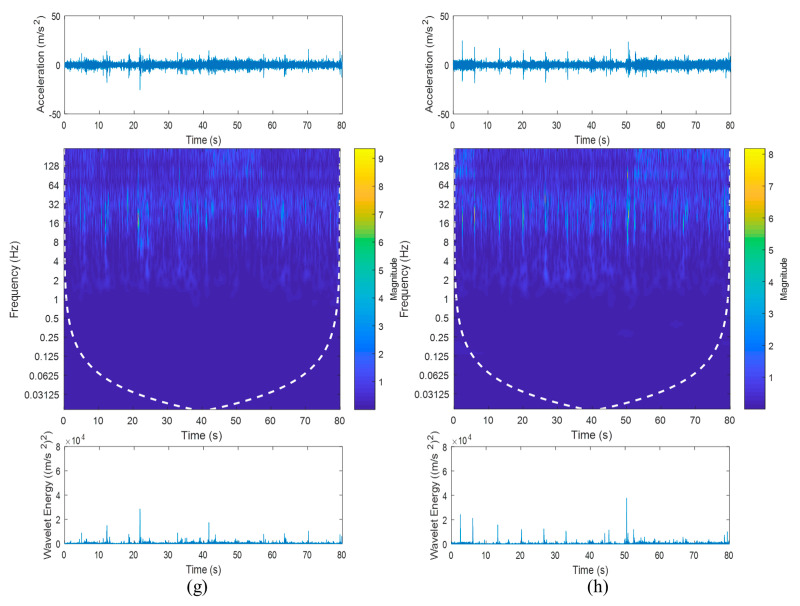
Time–frequency properties and wavelet energy of the vibration signals acquired from (**a**) Rural 1, (**b**) Rural 2, (**c**) Campus 1, (**d**) Campus 2, (**e**) Industrial 1, (**f**) Industrial 2, (**g**) Highway 1, and (**h**) Highway 2 road conditions.

Figure 5 shows the time–frequency properties and the wavelet energy of the vibration signals obtained through the CWT analysis. It can be confirmed that high-amplitude cycles had resulted in a high magnitude of wavelet coefficients. For example, the signals collected on rural roads had the highest magnitude of wavelet coefficients in the CWT mapping, which resulted in certain sections of the signals having high wavelet energy. This strongly indicates that CWT analysis is effective in detecting the high-amplitude events, which can cause high fatigue damage. Hence, wavelet transform can be applied to extract fatigue-related features from the vibration signals for fatigue life prediction.

### 3.2. Wavelet-Based Fatigue Features of Vibration Signals

This section discusses the results of the determination of low-frequency energy and multifractality of the vibration signals using wavelet-based approaches for the durability prediction of coil springs.

#### 3.2.1. Low-Frequency Vibration Energy Based on Hölder Singularities

The singularities in the vibration signals were determined using Hölder exponents and reconstructed into vibration signals. Statistical parameters of the reconstructed signals were determined and compared with the original signals. Table 2 lists the statistical parameters of the vibration signals reconstructed from the Hölder singularities and the differences between the original and reconstructed signals. The largest difference of 6.1% in RMS value was observed in the ‘Campus 1’ signal. The largest difference of 17.6% in skewness value was observed in the ‘Campus 2’ signal, which was due to the very small base values. The largest difference of 7.1% in kurtosis value was observed in the ‘Rural 2’ signal. As the differences in RMS and kurtosis were less than 10%, it can be said that the reconstructed signals had similar behaviours to the original signals.

**Figure 6:**
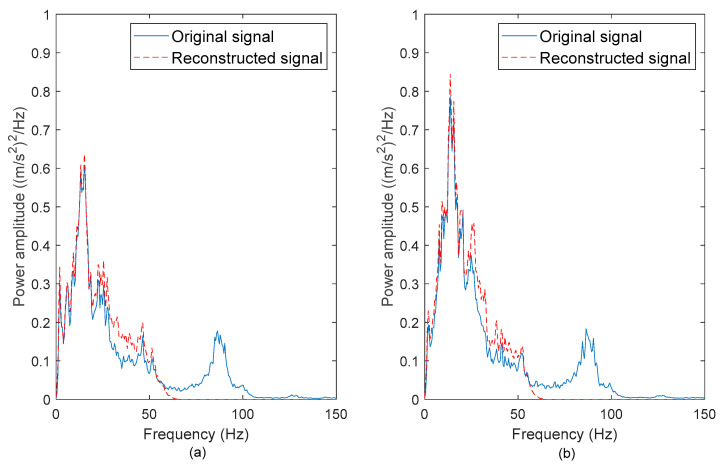
PSDs of the original and reconstructed vibration signals: (**a**) Rural 1 and (**b**) Rural 2.

**Figure 7:**
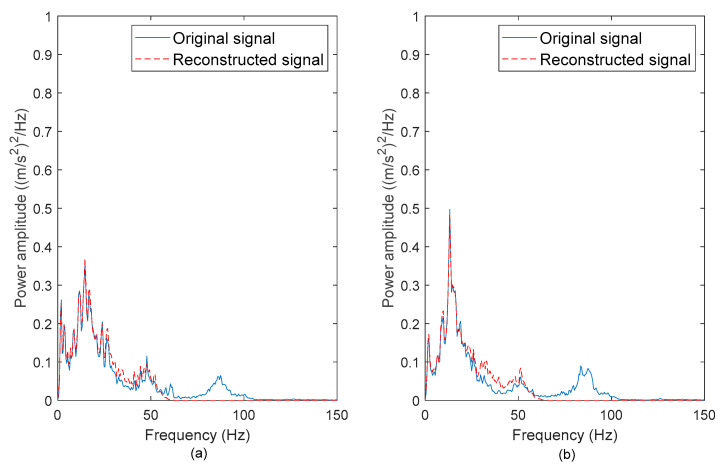
PSDs of the original and reconstructed vibration signals: (**a**) Campus 1 and (**b**) Campus 2.

**Figure 8:**
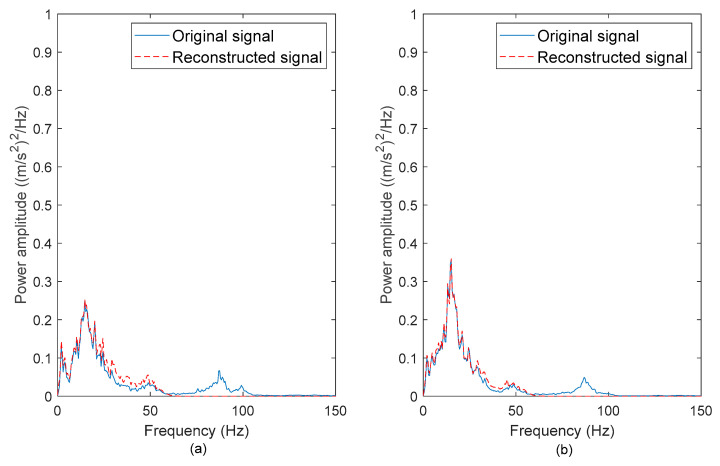
PSDs of the original and reconstructed vibration signals: (**a**) Industrial 1 and (**b**) Industrial 2.

**Figure 9:**
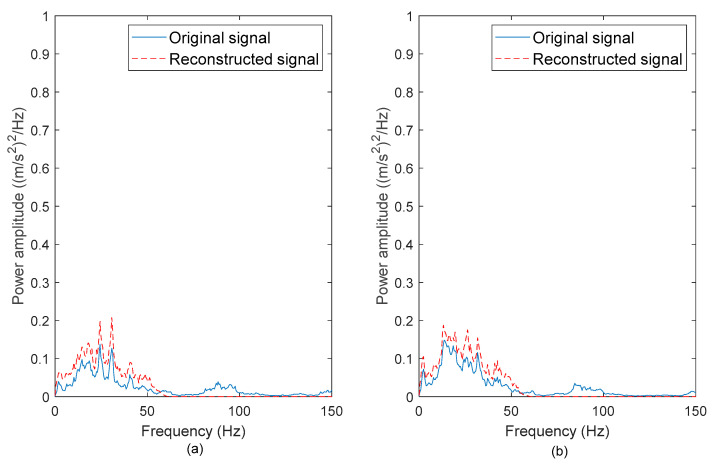
PSDs of the original and reconstructed vibration signals: (**a**) Highway 1 and (**b**) Highway 2.

To determine if the high-frequency noises were successfully removed, the PSDs of the original and reconstructed vibration signals were computed. Figure 6, Figure 7, Figure 8 and Figure 9 show the comparison between the PSDs of the original and reconstructed vibration signals obtained from the road tests conducted in rural areas, on the campus ground, in industrial areas, and on the highway, respectively. The PSD energy that represented the signal energy was subsequently computed, as listed in Table 3. After comparing the PSDs of the original and reconstructed signals, the high-frequency noises between 80–100 Hz in the original signals were found to have been eliminated. These high-frequency noises resulted from low-amplitude cycles that brought limited fatigue damage. The reconstructed signals matched the frequency spectrum of original signals between 0 to 50 Hz. This is in agreement with the previous study [42], in which the authors stated that road excitations are classified as low-frequency excitations within 0–50 Hz. The findings suggested that the singularity method can effectively remove the high-frequency noises, which is important to ensure the accuracy of the energy characterisation.

The PSDs of the ‘Rural’ signals exhibited the highest energy among the signals. This is because the ‘Rural’ signals contained had high amplitude ranges. Meanwhile, the signal energy of the ‘Highway’ signals was the least due to the smooth surface profile of the highway, which caused mild excitations. The highest PSD energy difference of 9.2% was observed in the ‘Campus 2’ signal. This proves that the energy in the vibration signals is mainly contributed by the low-frequency cycles. Therefore, the low-frequency energy can be a fatigue feature for the coil spring durability prediction.

#### 3.2.2. Multifractality of Vibration Signals

**Figure 10:**
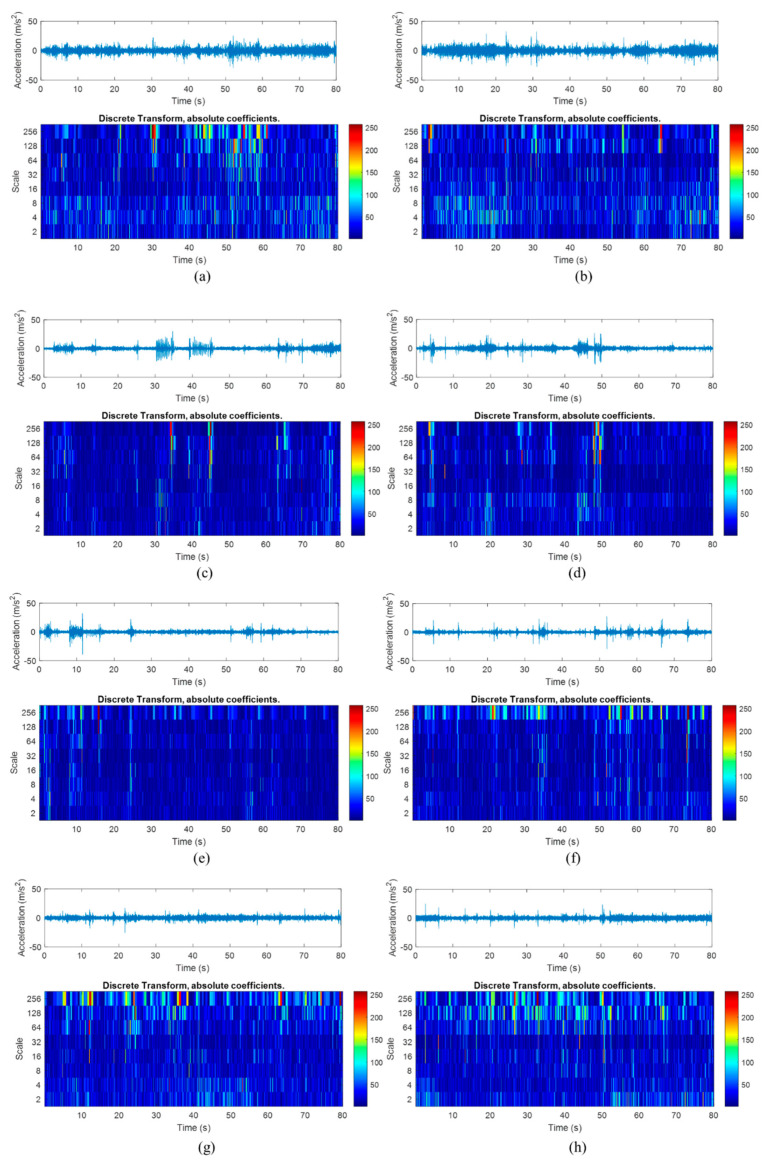
DWT wavelet coefficients of the vibration signals acquired under (**a**) Rural 1, (**b**) Rural 2, (**c**) Campus 1, (**d**) Campus 2, (**e**) Industrial 1, (**f**) Industrial 2, (**g**) Highway 1, and (**h**) Highway 2 road conditions.

DWT was applied to obtain the wavelet coefficients of the vibration signals, as illustrated in Figure 10. The DWT projected the high-amplitude events in the vibration loading as large-magnitude wavelet coefficients. In this study, the high-magnitude coefficients were the results of DWT performed on the low-frequency or high-scale signals, which is in agreement with the results of the PSD analysis. Unlike CWT, DWT requires less computational load due to the discretised properties, and hence, the WL method has the least computational load compared to other multifractal formalisms.

**Figure 11:**
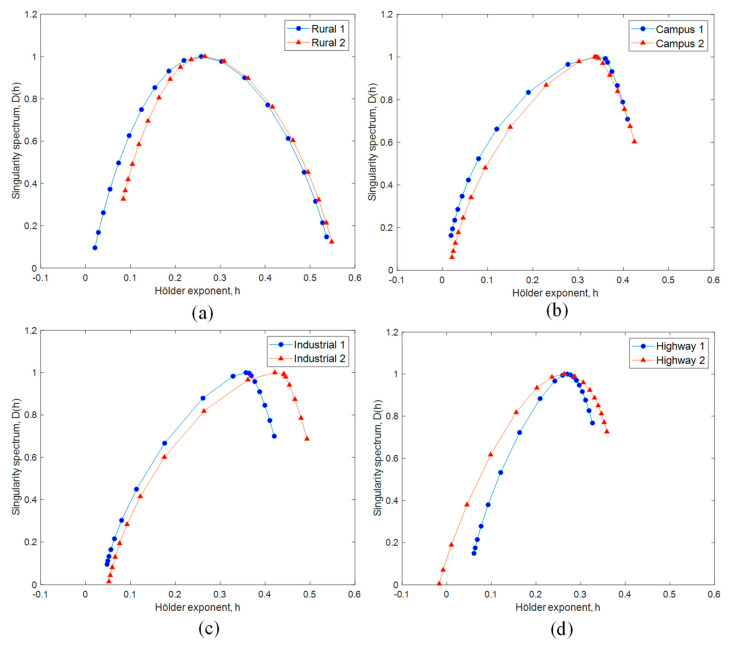
Multifractal spectrums of vibration signals under various road conditions: (**a**) rural road, (**b**) university campus road, (**c**) industrial road, and (**d**) highway.

**Figure 12:**
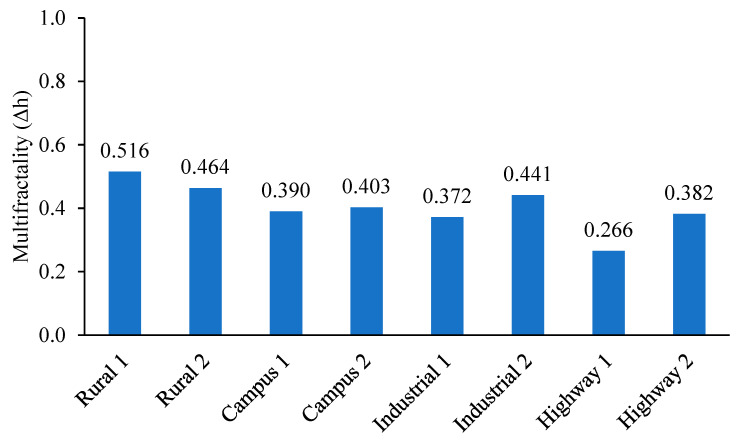
Multifractality of vibration signals computed from multifractal spectrums.

Multifractal spectrums of vibration were determined using the WL method, as illustrated in Figure 11. Due to the large spectrum width, the vibration signals were categorised as multifractal signals. Quan et al. [13] also supported that the road surfaces exhibited obvious multifractal properties. The multifractality of the vibration signals was computed from the spectrum width. Figure 12 shows the multifractality difference between the vibration signals. The signal with the highest multifractality of 0.516 was ‘Rural 1’. On the other hand, the ‘Highway 1’ signal had the least multifractality of 0.266. The multifractality characterises the complexity of a signal due to multiple scaling properties of a multifractal signal [43]. This is associated with the surface irregularities such as bumps and potholes on the road surfaces [13]. In this study, the unpaved and highly irregular surface of rural roads resulted in highly multifractal loading signals. Considering the close correlation between the loading multifractal properties and road surface roughness, the multifractality was chosen as the fatigue-related parameter for the fatigue life assessment.

### 3.3. Strain Signal Characterisation and Fatigue Life Assessment

**Figure 13:**
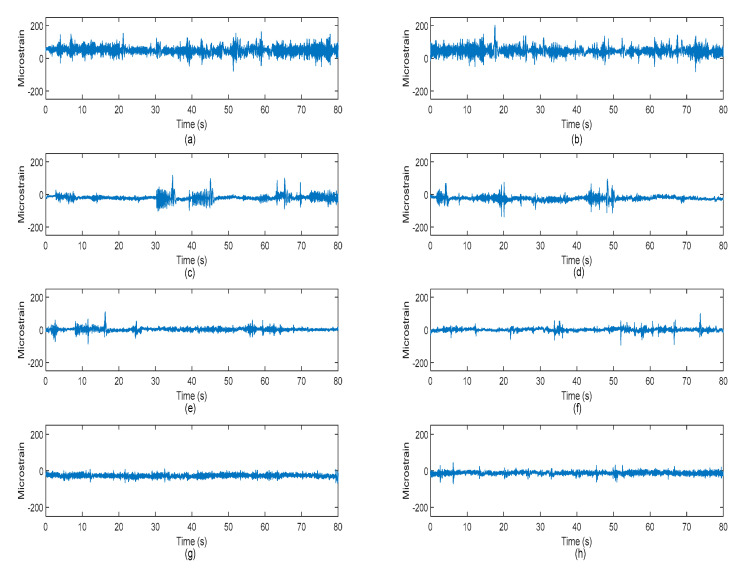
Strain histories acquired under (**a**) Rural 1, (**b**) Rural 2, (**c**) Campus 1, (**d**) Campus 2, (**e**) Industrial 1, (**f**) Industrial 2, (**g**) Highway 1, and (**h**) Highway 2 road conditions.

Strain histories were collected simultaneously with the vibration loading, and they represented the responses of the coil spring subjected to random road excitations. Figure 13 shows the strain loading histories of the coil spring collected from different roads. The statistical properties of the measured strain were investigated and listed in Table 4. The mean of the strain signal signified the loading condition (tensile or compressive) of the coil spring. The ‘Rural’ signals were tensile as the mean values were positive. In contrast, the ‘Campus’ and ‘Highway’ signals were compressive, while the ‘Industrial’ signals had zero-mean properties. Tensile loading is known to contribute more damage to the structure compared to compressive loading. The RMS values of the strain signals, which represented the strain energy, were closely related to high-amplitude events. In this work, the highest RMS of 53.45 µε was computed from the ‘Rural 1’ signal. The signals also had nearly symmetrical data distributions as the skewness values were close to zero, except for the ‘Campus 1’ and ‘Campus 2’ signals. Both of these signals had skewness values larger than one, indicating skewed-to-right distribution. This indicates that most of the cycles occurred below the mean level. As the kurtosis values were higher than three, it was confirmed that the strain signals exhibited non-stationary behaviours.

The fatigue life of the coil spring was determined from the strain histories with strain-life models. Table 5 shows the fatigue lives of the coil spring determined using the Coffin–Manson, Morrow, and SWT models. Overall, the ‘Rural’ signals contributed to the lowest fatigue life in the range of 10^3^–10^4^ cycles, while the longest fatigue life of 10^8^–10^9^ cycles was recorded in the ‘Highway’ signals. The former resulted from the large amplitude cycles in the ‘Rural’ signals, which caused high fatigue damage and shorter fatigue life. Overall, the Coffin–Manson model predicted longer fatigue lives compared to other models. This was because the Coffin–Manson model did not take the mean stress effects into account, which were likely to cause more fatigue damage [19]. Therefore, it is necessary to consider the mean stress effect when using the Morrow and SWT models.

### 3.4. Establishment of Vibration-Based Durability Prediction Model

**Figure 14:**
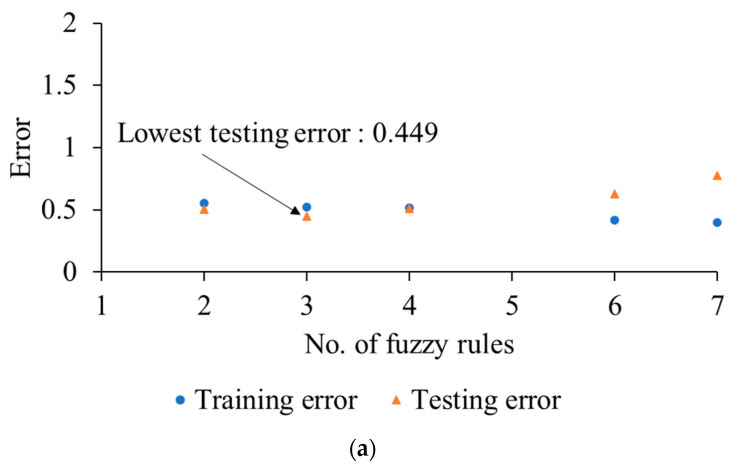

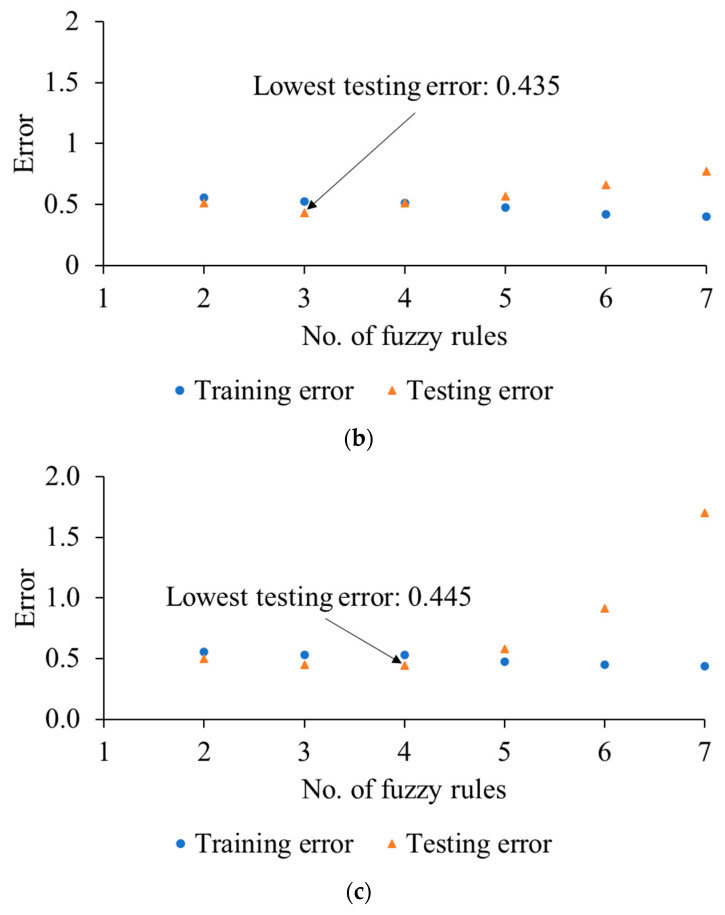
Training and testing errors of ANFIS models based on (**a**) Coffin–Manson, (**b**) Morrow, and (**c**) SWT fatigue life data.

In this study, a two-inputs-one-output ANFIS model for durability predictions was developed with low-frequency energy and multifractality as the inputs. The output of the model was the fatigue life of the coil spring. First, the number of fuzzy rules in the ANFIS model was optimised as this was necessary to obtain an optimum ANFIS structure. This was done by evaluating the accuracy of the trained ANFIS models by calculating the MSE of the predicted fatigue lives. The MSEs based on the training data were the training error, while MSEs of test data were the testing error. Figure 14 shows the training and testing errors of ANFIS models with various fuzzy rule numbers. The training errors of all ANFIS models exhibited a decreasing trend with the increasing number of fuzzy rules, indicating good fitting of the model to the training data. However, the testing errors must be observed to prevent overfitting. An increase in testing errors indicates that the model is overfitted. As seen in Figure 14, the testing errors for the Coffin–Manson- and Morrow-based ANFIS models increased after three fuzzy rules. Meanwhile, the testing errors for the SWT-based model increased after four fuzzy rules. Based on these findings, it was assumed that a further increase in the number of fuzzy rules would result in the overfitting of the models [35]. Therefore, the optimum number of fuzzy rules for the Coffin–Manson- and Morrow-based models was three, while for the SWT-based model, it was four.

**Figure 15:**
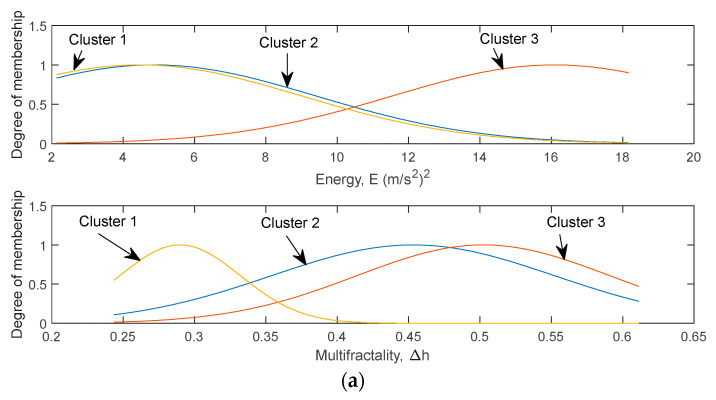

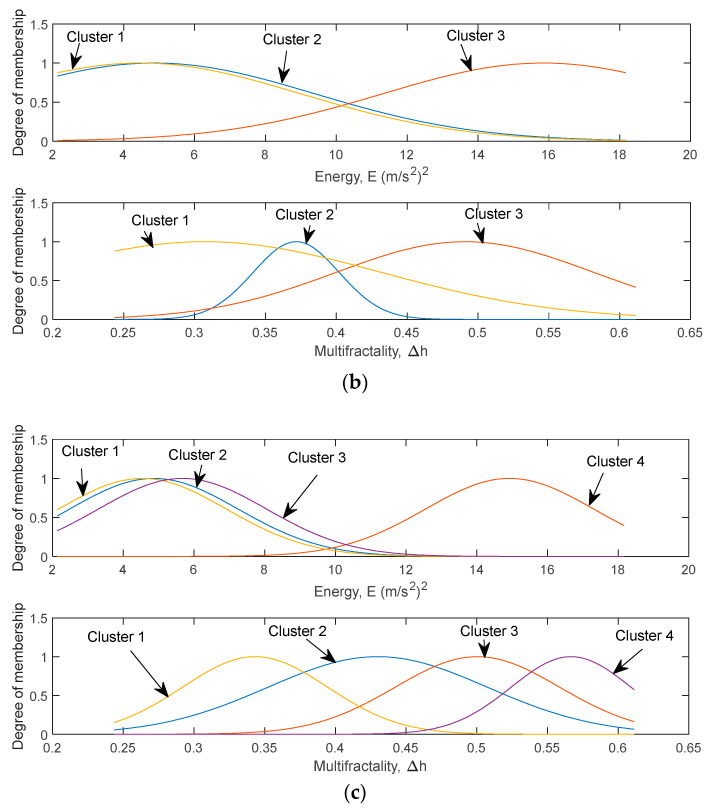
Optimised membership functions of input parameters for ANFIS models based on fatigue life data obtained using (**a**) Coffin–Manson, (**b**) Morrow, and (**c**) SWT models.

Figure 15 shows the optimised MFs of the input parameters of ANFIS models after the training process was completed. For the Coffin–Manson-based ANFIS model, the low-frequency energy and multifractality were characterised by three MFs, which were named according to the order of their mean values. The Morrow-based model also had a similar cluster number of the input variables as the Coffin–Manson-based model. It can be seen that the MF clusters 1 and 2 of the low-frequency signal energy were very close to each other. This was due to the highly dense input data distribution within the 2–10 (m/s^2^)^2^ range. For multifractality, three MFs were enough to cover the entire data range evenly. The SWT-based fatigue lives had a more complex relationship with the input variables, and thus, more fuzzy rules were needed to represent the SWT-based ANFIS model. It can be observed that both low-frequency energy and multifractality had four MFs. The first three clusters of low-frequency energy were close to each other, while the fourth cluster was located in the high-energy range. For multifractality, four MF clusters represented the multifractality of the loading signal at different levels.

**Figure 16:**
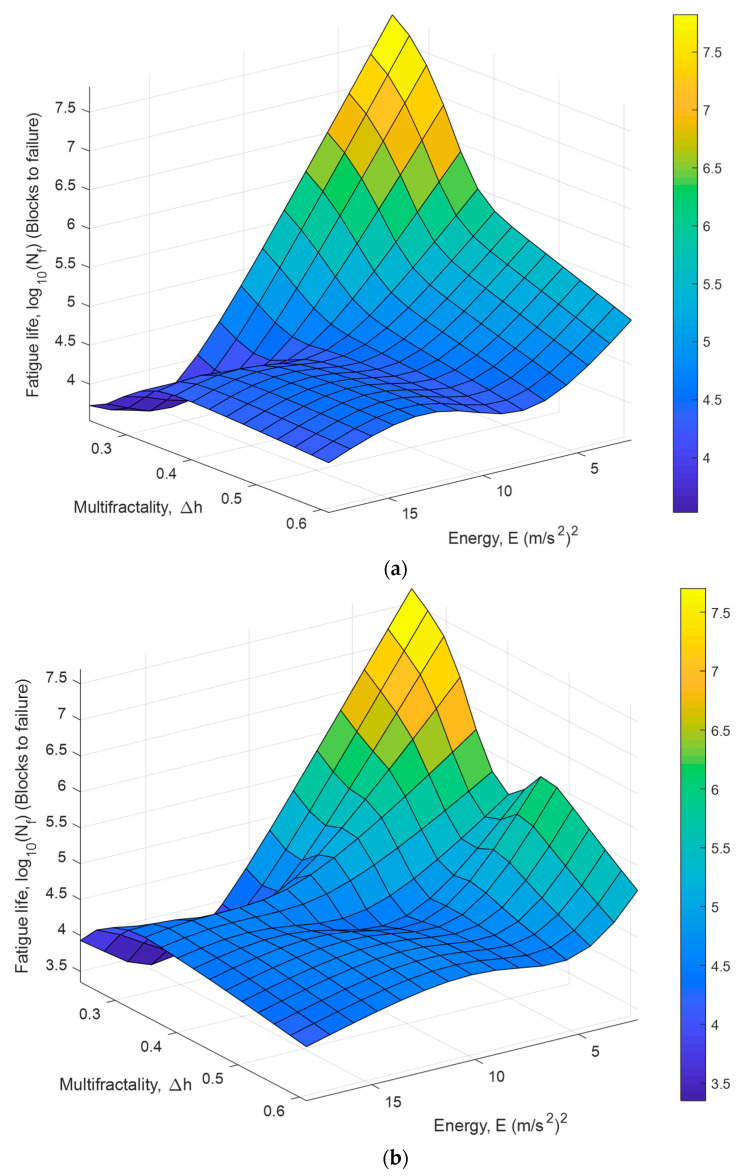

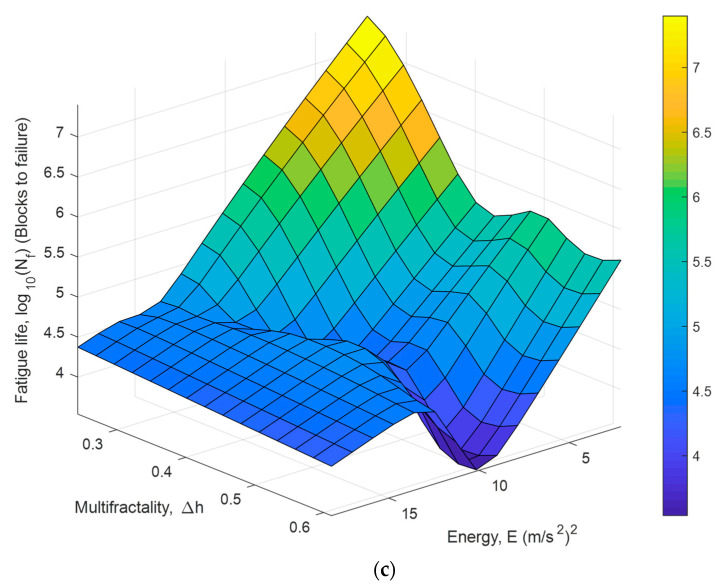
Response surface of trained ANFIS models based on fatigue life data obtained using (**a**) Coffin–Manson, (**b**) Morrow, and (**c**) SWT models.

Figure 16 shows the response surface plots predicted by the ANFIS models based on the multifractality and low-frequency energy of the vibration signals. Highly non-linear relationships between the fatigue life of the coil spring and the vibrational features were confirmed. The ANFIS models predicted the longest fatigue life of the coil spring under a low-energy and low-multifractality vibration load; for example, the ‘Highway’ signals. Farrahi et al. [44] stated that fatigue failures in automotive components are mainly related to road conditions. Bumpy roads such as those in rural and industrial areas are likely to produce loading signals with high multifractality [13]. Furthermore, surface irregularities also contribute to high-amplitude events with high energy. Therefore, the low-frequency energy and multifractality of the vibration signals significantly affect the durability performance of the coil springs and, thus, are suitable to be fatigue life prediction parameters.

### 3.5. Fatigue Life Comparison between ANFIS Models and Strain-Life Models

**Figure 17:**
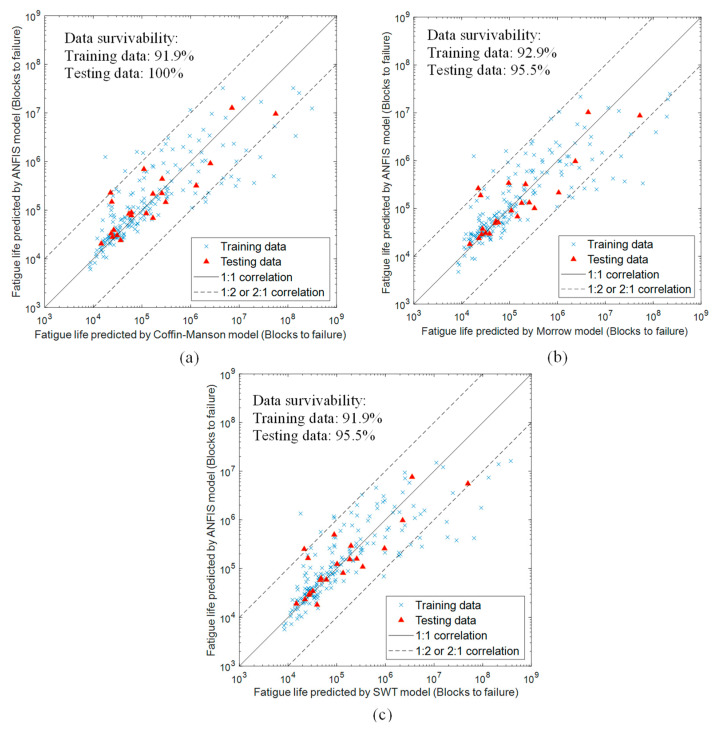
Fatigue life conservativity analysis of trained ANFIS models based on fatigue life data obtained using (**a**) Coffin–Manson, (**b**) Morrow, and (**c**) SWT models.

Fatigue life conservativity analysis was performed by comparing the fatigue lives predicted by the trained ANFIS models with those predicted by the corresponding strain-life models, as shown in Figure 17. The results revealed good conservativity of fatigue life predictions using the ANFIS models with more than 90% of the fatigue life data scattered within the 1:2 and 2:1 correlation boundaries. Furthermore, more than 95% of testing data survivability was observed, indicating good adaption of the trained ANFIS models to fresh datasets. However, some over-conservative fatigue life predictions to failure were also reported in a high-fatigue cycle regime above 10^6^ blocks [20]. The main reason can be related to the highly non-linear fatigue behaviours in the high-fatigue cycle regime, which eventually affect the fatigue life prediction in this region. Nevertheless, this issue did not limit the suitability of the models for durability prediction in a high-fatigue cycle regime as the accuracy level was still acceptable.

**Figure 18:**
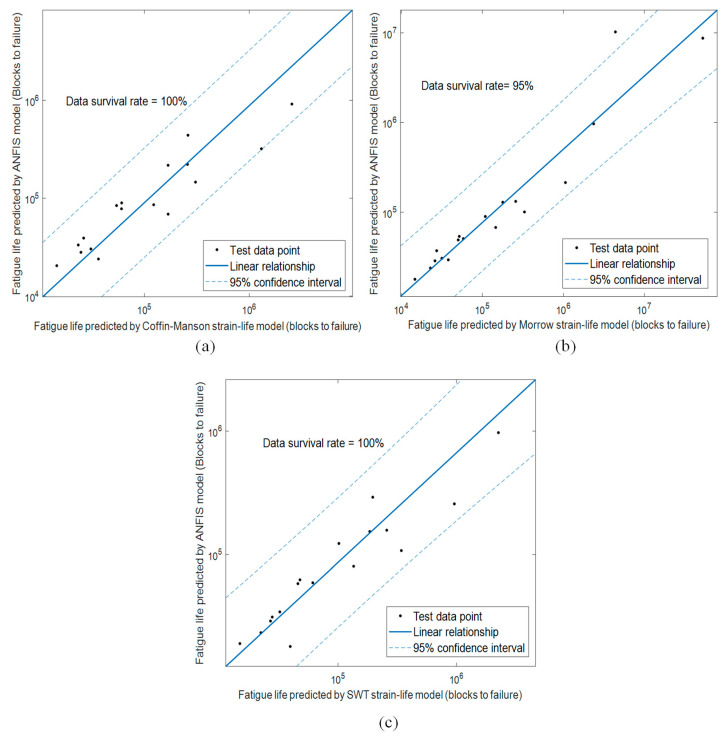
Fatigue life data survivability analysis within 95% confidence interval of ANFIS models based on (**a**) Coffin–Manson, (**b**) Morrow, and (**c**) SWT models.

**Figure 19:**
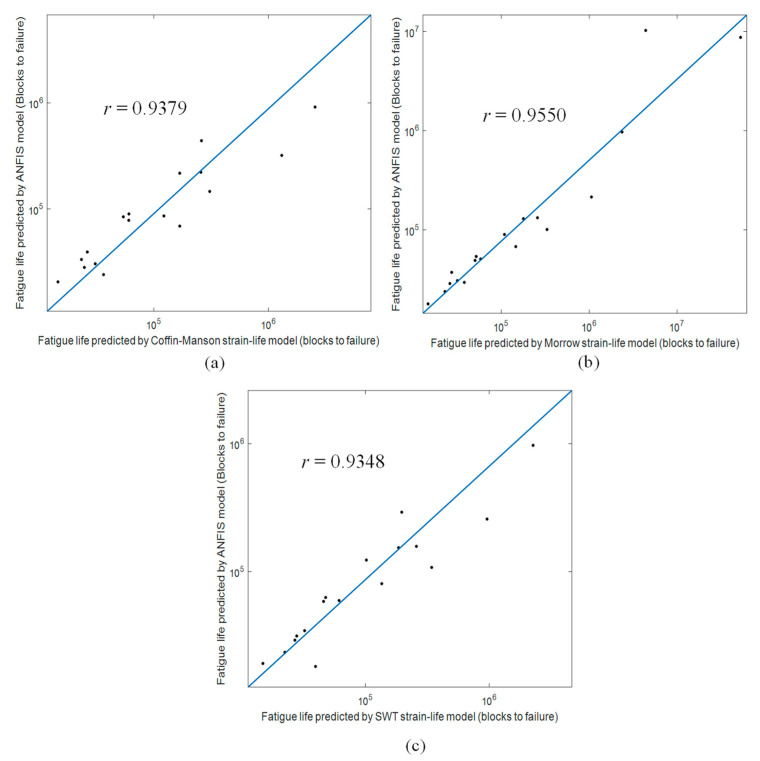
Fatigue life correlation analysis of ANFIS models with Pearson coefficients based on (**a**) Coffin–Manson, (**b**) Morrow, and (**c**) SWT models.

In addition, the 95% confidence interval data survivability analysis was also conducted. Figure 18 shows the correlation between the fatigue lives predicted using ANFIS models and strain-life models. This analysis was performed using the testing dataset for a more meaningful representation of the general use of the models. The results confirmed excellent data survivability above 95% and provided probabilistic validation to the acceptability of fatigue life predictions using the trained ANFIS models. Meanwhile, the correlation between the fatigue lives predicted using ANFIS models and strain-life models was represented by the Pearson coefficients (*r*). Figure 19 shows the linear correlation between the fatigue life data and the *r* values of each ANFIS model. The ANFIS models had *r* values higher than 0.9, indicating that the established ANFIS models were highly correlated to the strain-life models. Hence, it can be said that the established ANFIS models can accurately predict the fatigue life of coil springs using wavelet-based vibrational features. This can effectively reduce the dependency on strain data measurements and improve the efficiency of durability prediction of coil springs.

**Figure 20:**
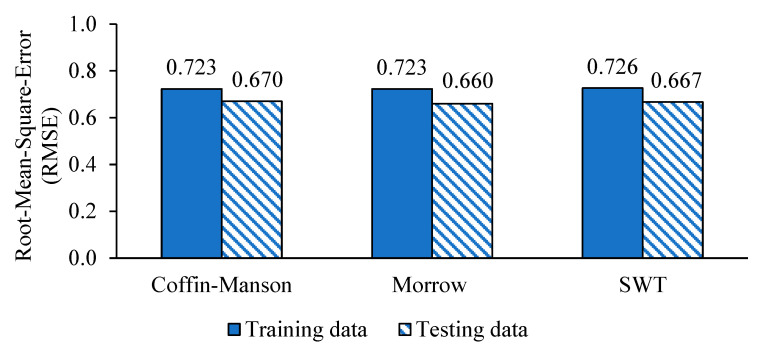
Root-mean-square-errors of ANFIS models based on the training and testing fatigue datasets.

The root-mean-square-errors (RMSE) of the predicted fatigue lives were calculated and are presented in Figure 20. It can be seen that the Coffin–Manson-, Morrow-, and SWT-based ANFIS models had similar accuracy as there were only slight differences between their RMSEs. The Morrow model had the least testing error of 0.660, and this finding was in agreement with the highest *r* value of 0.955 obtained by the model. Therefore, the Morrow model was recommended as the most suitable model for durability prediction of coil springs based on vibration data.

## 4. Conclusions

This study proposed a vibration-based durability prediction model for coil springs under random loading. The wavelet-based singularity and multifractal analysis were proposed to determine the low-frequency energy and multifractality of the vibration loading. The vibration features were associated with the durability of coil springs using the ANFIS method. Through Hölder singularities, the high-frequency noises were removed from the vibration signals, and this improved the accuracy of the signal energy analysis. The signal PSD energy had a close relationship with the high-amplitude events in the vibration signals, which contributed to high fatigue damage. Furthermore, the multifractal analysis revealed that the vibration signals possessed significant multifractal properties that were closely related to the signal complexity and road surface conditions. Hence, it can be confirmed that both vibration parameters could be used to characterise the road conditions and are suitable to be used as durability prediction parameters for coil springs. The results showed that the rural road loading signals caused lowest fatigue lives of 10^3^–10^4^ blocks to failure, while the longest fatigue lives of 10^3^–10^4^ blocks to failure were recorded in highway signals. The rural road signals with low fatigue lives were found to have the highest signal energy of 14–17 (m/s^2^)^2^ due to the large amplitude cycles in rural signals. Meanwhile, the highest multifractality of 0.4–0.5 were also recorded in the rural signals, mainly caused by the rough surface profile on the rural road. The established model provided fatigue life conservative with data survivability of more than 90% within the acceptable boundary. The models also had good fatigue data survivability within a 95% confidence interval and good correlation with *r* values higher than 0.9. The Morrow-based ANFIS model was recommended as the most suitable model for durability prediction of coil springs as it recorded the highest *r* value of 0.955 and the lowest RMSE of 0.660. This proposed model can be a better coil spring fatigue life prediction method as it only requires vibration data, and this reduces the dependency on strain data measurement for fatigue life assessment.

## Figures and Tables

**Table 1:** Statistical behaviour of vibration loading under different road conditions.

Road Condition	RMS (m/s^2^)	Skewness	Kurtosis
Rural 1	3.65	−0.01	5.56
Rural 2	4.31	0.01	5.87
Campus 1	2.77	0.03	12.11
Campus 2	2.56	−0.14	22.32
Industrial 1	2.34	−0.31	30.98
Industrial 2	2.19	0.02	21.54
Highway 1	2.14	−0.22	8.17
Highway 2	2.28	0.17	8.22

**Table 2:** Statistical parameters of the reconstructed vibration signals and the differences between original and reconstructed signals.

Road Condition	RMS		Skewness		Kurtosis	
	Value	Difference (%)	Value	Difference (%)	Value	Difference (%)
Rural 1	3.64	0.3	−0.01	0.0	5.80	4.3
Rural 2	4.00	7.2	0.01	0.0	5.45	7.1
Campus 1	2.60	6.1	−0.07	16.7	12.01	0.8
Campus 2	2.50	4.5	−0.15	17.6	21.14	5.2
Industrial 1	2.19	2.3	−0.36	16.1	29.00	6.4
Industrial 2	2.22	1.4	0.02	0.0	20.87	3.1
Highway 1	2.13	0.5	−0.22	0.0	8.01	2.0
Highway 2	2.24	1.8	0.16	5.8	7.96	3.2

**Table 3:** Comparison of PSD energy before and after signal reconstruction.

Road Condition	PSD Energy (m/s^2^)^2^		Percentage of Differences (%)
	Original Signal	Reconstructed Signal	
Rural 1	14.36	13.36	7.0
Rural 2	16.95	16.00	5.6
Campus 1	7.35	6.79	7.7
Campus 2	6.95	6.31	9.2
Industrial 1	5.02	4.78	4.7
Industrial 2	5.25	4.98	5.3
Highway 1	4.42	4.47	0.9
Highway 2	4.95	5.00	1.1

**Table 4:** Statistical behaviours of strain loading histories.

Road Condition	Mean (µε)	RMS (µε)	Skewness	Kurtosis
Rural 1	47.69	53.45	0.19	6.53
Rural 2	44.33	48.78	0.54	7.22
Campus 1	−16.71	25.18	1.57	21.24
Campus 2	−25.51	29.10	1.22	20.88
Industrial 1	2.56	19.77	0.75	13.12
Industrial 2	0.78	19.35	0.33	11.67
Highway 1	−25.79	16.73	0.09	3.54
Highway 2	−13.27	13.51	0.11	3.12

**Table 5:** Counted rainflow cycles and fatigue lives computed from strain loading histories using strain-life approaches.

Road Condition	Rainflow Cycles	Fatigue Life (Blocks to Failure)		
		Coffin–Manson	Morrow	Smith–Watson–Topper
Rural 1	4569	3.10 × 10^4^	2.49 × 10^4^	2.20 × 10^4^
Rural 2	4517	6.07 × 10^3^	5.35 × 10^3^	4.96 × 10^3^
Campus 1	7946	1.11 × 10^5^	1.07 × 10^5^	1.05 × 10^5^
Campus 2	7982	8.57 × 10^4^	1.15 × 10^5^	1.37 × 10^5^
Industrial 1	7994	3.69 × 10^5^	3.22 × 10^5^	3.00 × 10^5^
Industrial 2	8028	4.59 × 10^5^	4.38 × 10^5^	4.27 × 10^5^
Highway 1	8078	3.58 × 10^9^	2.29 × 10^9^	4.47 × 10^9^
Highway 2	8057	7.83 × 10^8^	1.79 × 10^8^	4.42 × 10^8^

## Data Availability

This study did not report any data.

## List of Nomenclature and Symbols

ψ(*t*)	Mother wavelet function
*s*	Scaling parameter
*u*	Shift parameter
$W_{\psi }f\left(s,u\right)$	Wavelet coefficient
*m*	Vanishing moment
$P\left(\omega \right)$	Power spectral densities (PSD)
ω	Frequency
*E_PSD_*	PSD energy
$\varepsilon_{a}$	Strain amplitude
*E*	Modulus of elasticity
$\sigma_{f}^{\prime }$	Fatigue strength coefficient
*b*	Fatigue strength exponent
$\varepsilon_{f}^{\prime }$	Fatigue ductility coefficient
c	Fatigue ductility exponent
$\sigma_{max}$	Maximum stress amplitude
$\sigma_{a}$	Stress amplitude
2*N_f_*	Load reversals to failure
$D_{i}$	Fatigue damage caused by one fatigue cycle
D	Total fatigue damage
*N_fi_*	Cycles to failure
*n_i_*	Counted fatigue cycles
$\sigma_{m}$	Mean stress
$P\left(\omega \right)$	Power spectral densities
HE	Hölder exponent
DOG	Derivative of Gaussian

## Appendix A

**Table A1:** Training dataset for ANFIS modelling.

No.	Energy (m/s^2^)^2^	Multifractality	Fatigue Life (Base-10 Logarithm Scale)		
			Coffin–Manson	Morrow	SWT
1	18.153	0.401	3.926	3.919	3.917
2	18.168	0.523	3.942	3.931	3.927
3	16.908	0.491	3.948	3.950	3.953
4	6.238	0.603	4.032	4.054	4.069
5	18.153	0.401	4.056	4.051	4.049
6	18.168	0.523	4.071	4.061	4.057
7	16.908	0.491	4.077	4.083	4.088
8	8.007	0.481	4.081	4.089	4.092
9	18.153	0.401	4.148	4.144	4.143
10	6.238	0.603	4.161	4.189	4.205
11	18.168	0.523	4.162	4.154	4.149
12	16.908	0.491	4.171	4.179	4.185
13	7.051	0.455	4.180	4.194	4.199
14	17.124	0.559	4.187	4.190	4.193
15	5.077	0.369	4.195	4.189	4.188
16	7.421	0.445	4.198	4.195	4.190
17	16.084	0.504	4.199	4.187	4.179
18	14.928	0.503	4.208	4.217	4.225
19	18.153	0.401	4.217	4.214	4.212
20	18.168	0.523	4.231	4.223	4.218
21	16.908	0.491	4.242	4.252	4.257
22	4.517	0.318	4.249	4.252	4.258
23	6.238	0.603	4.256	4.288	4.304
24	15.338	0.490	4.268	4.274	4.278
25	15.838	0.493	4.292	4.292	4.294
26	7.421	0.445	4.313	4.308	4.302
27	14.126	0.579	4.317	4.328	4.337
28	14.711	0.486	4.318	4.331	4.342
29	5.077	0.369	4.319	4.313	4.314
30	6.238	0.603	4.329	4.363	4.378
31	13.083	0.514	4.335	4.345	4.350
32	4.831	0.533	4.341	4.332	4.327
33	14.928	0.503	4.349	4.364	4.373
34	6.183	0.401	4.356	4.364	4.374
35	14.608	0.579	4.357	4.363	4.370
36	5.222	0.425	4.369	4.357	4.344
37	14.715	0.525	4.370	4.378	4.384
38	5.207	0.405	4.387	4.381	4.371
39	7.421	0.445	4.395	4.389	4.382
40	14.679	0.503	4.400	4.401	4.404
41	13.327	0.485	4.401	4.414	4.420
42	6.786	0.406	4.404	4.395	4.385
43	5.077	0.369	4.406	4.401	4.403
44	15.488	0.508	4.407	4.400	4.394
45	6.622	0.392	4.411	4.381	4.360
46	13.342	0.492	4.422	4.422	4.422
47	5.120	0.463	4.422	4.444	4.458
48	15.338	0.490	4.422	4.434	4.443
49	18.153	0.401	4.425	4.423	4.419
50	14.812	0.483	4.425	4.443	4.453
51	5.733	0.441	4.431	4.419	4.405
52	18.168	0.523	4.437	4.428	4.421
53	14.086	0.527	4.442	4.438	4.437
54	15.127	0.455	4.445	4.453	4.457
55	14.928	0.503	4.455	4.472	4.483
56	13.646	0.534	4.456	4.447	4.445
57	7.421	0.445	4.458	4.450	4.443
58	14.483	0.478	4.458	4.460	4.462
59	16.908	0.491	4.459	4.469	4.474
60	12.921	0.611	4.465	4.464	4.468
61	5.077	0.369	4.469	4.466	4.470
62	15.379	0.476	4.490	4.513	4.527
63	13.832	0.515	4.492	4.488	4.487
64	4.831	0.533	4.508	4.500	4.495
65	5.675	0.561	4.512	4.530	4.541
66	8.072	0.392	4.525	4.533	4.539
67	4.253	0.384	4.527	4.542	4.558
68	14.928	0.503	4.537	4.537	4.568
69	15.338	0.490	4.537	4.554	4.565
70	13.171	0.468	4.542	4.547	4.552
71	6.238	0.603	4.551	4.584	4.593
72	6.622	0.392	4.551	4.512	4.488
73	4.706	0.523	4.564	4.566	4.569
74	9.076	0.441	4.589	4.601	4.615
75	6.312	0.416	4.591	4.589	4.586
76	8.536	0.467	4.592	4.581	4.572
77	15.245	0.469	4.603	4.611	4.614
78	15.338	0.490	4.627	4.647	4.659
79	5.199	0.453	4.629	4.606	4.590
80	7.732	0.485	4.630	4.618	4.606
81	4.831	0.533	4.635	4.627	4.622
82	7.421	0.445	4.651	4.637	4.628
83	5.077	0.369	4.654	4.660	4.671
84	6.622	0.392	4.656	4.610	4.585
85	5.195	0.408	4.703	4.686	4.672
86	18.153	0.401	4.703	4.700	4.695
87	18.168	0.523	4.709	4.702	4.695
88	4.831	0.533	4.734	4.726	4.722
89	6.160	0.472	4.736	4.676	4.644
90	6.622	0.392	4.737	4.687	4.660
91	16.908	0.491	4.749	4.763	4.769
92	5.652	0.391	4.749	4.778	4.798
93	5.791	0.445	4.768	4.691	4.651
94	2.323	0.388	4.771	4.727	4.698
95	3.439	0.512	4.790	4.869	4.924
96	14.928	0.503	4.798	4.824	4.838
97	5.199	0.453	4.799	4.768	4.751
98	4.781	0.371	4.813	4.867	4.909
99	4.866	0.427	4.825	4.850	4.878
100	6.238	0.603	4.830	4.841	4.836
101	5.256	0.438	4.839	4.875	4.912
102	4.617	0.453	4.857	4.818	4.792
103	6.379	0.453	4.875	4.873	4.870
104	5.077	0.369	4.897	4.926	4.951
105	5.332	0.508	4.906	4.858	4.830
106	6.573	0.496	4.911	4.896	4.886
107	15.338	0.490	4.914	4.945	4.963
108	7.421	0.445	4.924	4.900	4.886
109	2.323	0.388	4.951	4.889	4.854
110	4.953	0.498	4.970	4.907	4.872
111	3.439	0.512	4.972	5.064	5.126
112	6.622	0.392	4.994	4.932	4.901
113	2.964	0.339	5.021	4.981	4.956
114	2.133	0.357	5.023	4.970	4.941
115	5.199	0.453	5.025	4.986	4.966
116	4.617	0.453	5.036	4.991	4.963
117	2.267	0.388	5.044	4.976	4.940
118	2.531	0.402	5.047	4.982	4.948
119	4.057	0.424	5.047	5.002	4.976
120	7.132	0.375	5.051	5.003	4.974
121	4.247	0.441	5.051	5.003	4.974
122	4.831	0.533	5.052	5.045	5.040
123	4.976	0.462	5.080	5.022	4.988
124	5.199	0.453	5.088	5.036	5.007
125	2.323	0.388	5.097	5.017	4.978
126	3.439	0.512	5.106	5.208	5.276
127	3.922	0.381	5.137	5.141	5.143
128	4.617	0.453	5.168	5.120	5.090
129	14.928	0.503	5.184	5.217	5.233
130	4.024	0.458	5.187	5.134	5.104
131	3.439	0.512	5.206	5.318	5.392
132	7.581	0.356	5.207	5.139	5.101
133	2.323	0.388	5.217	5.122	5.077
134	2.964	0.339	5.236	5.183	5.153
135	4.617	0.453	5.272	5.220	5.190
136	4.798	0.383	5.289	5.318	5.336
137	3.922	0.381	5.329	5.335	5.339
138	15.338	0.490	5.337	5.379	5.401
139	5.199	0.453	5.347	5.300	5.277
140	6.622	0.392	5.361	5.295	5.261
141	2.964	0.339	5.403	5.335	5.301
142	2.600	0.363	5.444	5.359	5.317
143	3.922	0.381	5.483	5.493	5.498
144	5.730	0.244	5.488	5.512	5.518
145	4.511	0.302	5.499	5.494	5.490
146	4.831	0.533	5.508	5.500	5.491
147	3.439	0.512	5.518	5.677	5.782
148	4.540	0.309	5.522	5.443	5.405
149	3.966	0.350	5.531	5.471	5.442
150	4.131	0.377	5.533	5.531	5.529
151	2.964	0.339	5.538	5.457	5.418
152	4.617	0.453	5.609	5.542	5.508
153	3.922	0.381	5.609	5.622	5.629
154	2.323	0.388	5.632	5.479	5.418
155	4.511	0.302	5.690	5.682	5.676
156	4.540	0.309	5.786	5.688	5.643
157	5.199	0.453	5.845	5.787	5.756
158	4.511	0.302	5.849	5.838	5.832
159	4.511	0.302	5.982	5.969	5.961
160	3.439	0.512	5.990	6.239	6.400
161	2.964	0.339	5.995	5.865	5.810
162	4.540	0.309	6.000	5.886	5.835
163	3.835	0.373	6.028	5.992	5.968
164	3.922	0.381	6.034	6.059	6.071
165	4.602	0.377	6.098	6.237	6.311
166	4.617	0.453	6.128	6.039	5.997
167	3.924	0.350	6.162	6.079	6.039
168	4.540	0.309	6.172	6.046	5.990
169	4.927	0.300	6.226	6.202	6.190
170	5.360	0.302	6.240	6.185	6.158
171	2.323	0.388	6.264	6.039	5.950
172	3.479	0.455	6.307	6.293	6.280
173	4.465	0.325	6.310	6.090	5.991
174	5.324	0.302	6.310	6.297	6.289
175	3.835	0.373	6.372	6.317	6.281
176	4.544	0.280	6.391	6.369	6.356
177	4.511	0.302	6.435	6.415	6.405
178	3.835	0.373	6.635	6.566	6.522
179	2.964	0.339	6.664	6.480	6.400
180	3.922	0.381	6.666	6.703	6.723
181	4.465	0.325	6.678	6.455	6.343
182	4.540	0.309	6.720	6.567	6.493
183	3.835	0.373	6.836	6.758	6.708
184	3.456	0.322	6.889	6.678	6.579
185	4.129	0.372	6.927	6.817	6.750
186	4.465	0.325	6.944	6.726	6.606
187	4.351	0.354	6.986	7.161	7.278
188	4.511	0.302	7.089	7.063	7.048
189	4.465	0.325	7.145	6.932	6.804
190	4.494	0.357	7.305	7.404	7.462
191	3.835	0.373	7.433	7.330	7.261
192	4.540	0.309	7.449	7.284	7.187
193	3.279	0.372	7.711	7.784	7.824
194	4.465	0.325	7.731	7.533	7.387
195	2.589	0.250	8.111	8.347	8.576
196	3.835	0.373	8.161	8.057	7.970
197	2.776	0.255	8.231	8.309	8.327
198	4.465	0.325	8.497	8.284	8.121

**Table A2:** Testing dataset for ANFIS modelling.

No.	Energy (m/s^2^)^2^	Multifractality	Fatigue Life (Base-10 Logarithm Scale)		
			Coffin–Manson	Morrow	SWT
1	5.189	0.359	4.355	4.345	4.332
2	5.622	0.423	4.734	4.765	4.787
3	2.244	0.421	5.416	5.334	5.293
4	2.187	0.385	5.041	4.981	4.949
5	4.238	0.540	5.226	5.165	5.132
6	4.867	0.469	5.088	5.036	5.007
7	5.686	0.430	4.782	4.702	4.661
8	5.506	0.422	4.783	4.715	4.678
9	2.692	0.300	7.749	7.721	7.696
10	2.923	0.283	6.850	6.642	6.549
11	4.359	0.330	6.407	6.373	6.355
12	3.802	0.380	6.116	6.026	5.982
13	4.145	0.396	5.226	5.252	5.268
14	4.114	0.395	5.411	5.412	5.412
15	5.002	0.393	5.488	5.520	5.535
16	7.765	0.440	4.369	4.359	4.348
17	8.562	0.481	4.562	4.580	4.596
18	5.816	0.363	4.380	4.395	4.411
19	5.675	0.535	4.420	4.436	4.445
20	14.953	0.488	4.394	4.415	4.430
21	13.745	0.524	4.489	4.500	4.508
22	16.600	0.560	4.164	4.168	4.171
